# Investigating pathological epigenetic aberrations by epi-proteomics

**DOI:** 10.1186/s13148-022-01371-y

**Published:** 2022-11-12

**Authors:** Giulia Robusti, Alessandro Vai, Tiziana Bonaldi, Roberta Noberini

**Affiliations:** 1 Department of Experimental Oncology, IEO, European Institute of Oncology IRCCS, 20139 Milan, Italy; 2 Department of Oncology and Hematology-Oncology, University of Milan, 20122 Milan, Italy

**Keywords:** Cancer, Epigenetics, Histone-modifying enzyme, Histone posttranslational modification, Histone variant, Mass spectrometry, Proteomics

## Abstract

Epigenetics includes a complex set of processes that alter gene activity without modifying the DNA sequence, which ultimately determines how the genetic information common to all the cells of an organism is used to generate different cell types. Dysregulation in the deposition and maintenance of epigenetic features, which include histone posttranslational modifications (PTMs) and histone variants, can result in the inappropriate expression or silencing of genes, often leading to diseased states, including cancer. The investigation of histone PTMs and variants in the context of clinical samples has highlighted their importance as biomarkers for patient stratification and as key players in aberrant epigenetic mechanisms potentially targetable for therapy. Mass spectrometry (MS) has emerged as the most powerful and versatile tool for the comprehensive, unbiased and quantitative analysis of histone proteoforms. In recent years, these approaches—which we refer to as “epi-proteomics”—have demonstrated their usefulness for the investigation of epigenetic mechanisms in pathological conditions, offering a number of advantages compared with the antibody-based methods traditionally used to profile clinical samples. In this review article, we will provide a critical overview of the MS-based approaches that can be employed to study histone PTMs and variants in clinical samples, with a strong focus on the latest advances in this area, such as the analysis of uncommon modifications and the integration of epi-proteomics data into multi-OMICs approaches, as well as the challenges to be addressed to fully exploit the potential of this novel field of research.

## Background

**Fig. 1:**
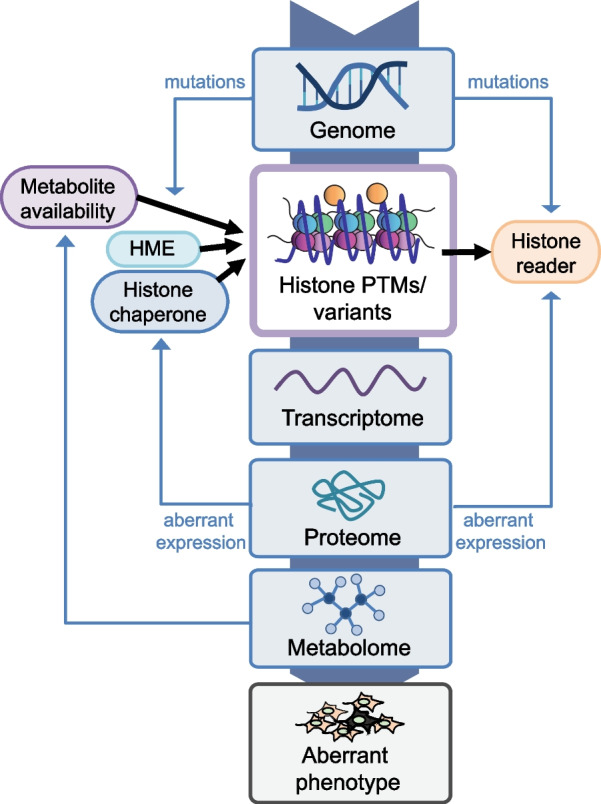
Role of histones in the regulation of gene expression. Histone posttranslational modifications (PTMs) and variants contribute to the regulation of the expression of genes, determining changes at the levels of transcripts, proteins and metabolites that can lead to aberrant phenotypes. In turn, proteins and metabolites can influence the levels and the effects of histone PTMs and variants, by affecting the levels of histone-modifying enzymes (HMEs), histone chaperones and readers, and intermediate metabolites

Epigenetics includes a complex set of processes that alter gene activity without modifying the DNA sequence, which ultimately defines cell fate by determining how shared genetic information is used to generate different phenotypes. Histones are part of the epigenetic machinery and contribute to two fundamental nuclear functions: DNA compaction and regulation of gene expression. Histones are small, basic proteins characterized by a C-terminal globular domain and an N-terminal tail. In the nucleus of eukaryotic cells, they are bound to DNA to form the nucleosome, the basic unit of the chromatin. Around 146 bp of DNA are wrapped around the so-called core histone octamer that consists of two copies of histone H2A and H2B, and a dimer of histone H3 and H4, while a linker histone H1 contributes to chromatin stabilization by binding the nucleosome and the linker DNA present between nucleosomes [1]. In addition to the canonical forms, variants of core and linker histones exist and play a role in the regulation of chromatin structure and gene expression [2]. Histones are decorated by a number of posttranslational modifications (PTMs), which occur mainly at their N-terminal tails and include methylation, acylation (the most abundant of which is mono-acetylation), phosphorylation, ubiquitylation, ADP-ribosylation, SUMOylation, deamination, as well as other less common modifications [3–5]. Histones contribute to DNA packaging within the nucleus, and thanks to the presence of different combinations of PTMs and variants, they contribute to the regulation of gene expression and cell fate. Histone PTMs are deposed and removed by a group of enzymes collectively known as histone-modifying enzymes (HMEs) and exert their downstream effects by binding to effector proteins called “readers” [6]. In addition, histone chaperones influence histone levels by transporting newly synthesized histones to specific sites in the genome [7]. Aberrations in the patterns of histone PTMs and variants can result in the inappropriate expression of genes, which causes altered transcript, protein and metabolite levels, ultimately leading to aberrant phenotypes (Fig. 1).

In the last decade, histone PTMs and variants have been investigated in a wide range of human diseases, including cancer, neurodegenerative diseases, heart failure, as well as autoimmune and infectious diseases. While genetic defects, such as mutations, deletions, or copy number changes, have been long considered the major contributors to cancer development and progression, epigenetics has emerged as an important player in various cancer-related processes [8]. For instance, the loss of H3K14ac, H4K20me3 and H4K16ac was reported as a common hallmark of cancer [9, 10], while other modifications—including acetylation, H3K4me2, H3K9me3, H3K27me3—or combinations of modifications, correlate with cancer patient prognosis [11], with effects that are context dependent, and can even go in opposite directions depending on the specific cancer type [12]. Histone PTMs also have diagnostic potential, particularly when measured from circulating nucleosomes, which are released in the blood following cell death and apoptosis [13], as demonstrated by studies detecting histone PTM patterns specific to the cancerous state in pancreatic and colorectal cancers [14, 15].

In addition to cancer, epigenetic modifications are emerging to have a key role in the development of other diseases. Histone PTMs have been described in the development of neurodegenerative disorders, characterized by continuous degeneration and death of nerve cells, many of which have no known genetic cause. Few studies have been performed in Alzheimer’s (AD) and Parkinson’s diseases, where a global increase in lysine acetylation was identified in diseased patients compared to healthy controls [16–18]. Emerging evidence suggests that acetylation and methylation of histones are involved also in the regulation of gene expression during the progression of cardiac hypertrophy, a pathological state characterized by increased cardiac myocyte size, excessive protein synthesis and the consequent development of heart failure, which is characterized by a specific epigenetic signature compared with healthy tissue [19]. The role of histone H3 methylation and acetylation in autoimmune diseases, including rheumatoid arthritis, lupus erythematosus and Type 1 (T1) diabetes was also investigated (reviewed in [20]). For instance, in T1 diabetes, ChIP-seq experiments of several histone acetylation and methylation marks revealed an association of H3K9 acetylation (H3K9Ac) with the expression of T1 diabetes susceptible genes [21]. In addition, a number of epigenetic changes occur as a direct result of Herpes Simplex Virus infection [22].

Alterations in the levels of many core and linker histone variants have also been linked to various diseases, particularly cancer (reviewed in [23]). For example, the histone variant H2A.z is known to be upregulated in many cancers (reviewed in [24]) as well in cardiac hypertrophy [25], while macro-H2A is a general tumor suppressor (although its role is context dependent) [26] and a prognostic marker for the development of Huntington disease [27, 28].

These studies underline the importance of studying histone PTMs in the context of disease, which has been carried out historically through methods based on the use of antibodies, such as immunoblots, immunohistochemistry (IHC) and enzyme-linked immunosorbent assay (ELISAs). Although these studies showed the potential of investigating histones, both for the discovery of biomarkers and epigenetic mechanisms potentially targetable for therapy, the use of antibody-based methods has a number of limitations, as discussed in more detail in the next sections. Such limitations can be overcome by employing mass spectrometry (MS), which has become the method of choice for the quantitative, unbiased and comprehensive profiling of histone proteoforms, namely histones containing different PTMs and histone variants. In recent years, these approaches—which we refer to as “epi-proteomics”—have demonstrated their usefulness for the investigation of epigenetics in pathological conditions. In this review, we will provide an overview on the recent MS-based strategies for the analysis of histones, their PTMs and their variants that can be applied to clinical samples. We will first describe the MS workflows that have been already implemented for the analysis of patient samples and highlight their contribution to our current knowledge of histone–mediated mechanisms in diseased states (results summarized in Table 1). We will then focus on the challenges to be addressed to fully exploit the potential of this novel field of research, also in the context of multi-OMICs platforms.

**Table 1:** Pathological epigenetic aberrations investigated by epi-proteomics

Disease	Type of sample^1^	Main findings of MS experiment	Technical improvement	MS method^2^	Quantification approach ^3^	Ref
Multiple cancers	Fresh frozen tissue	Loss of H4K20me3 and H4K16ac is a common hallmark of cancer		LC–MS, DDA	Label-free	[9]
	FFPE, resh/OCT-frozen tissue	Loss of H3K14ac is a common hallmark of cancer, additional tumor- and subtype-specific changes exist		LC–MS, DDA	Super-SILAC	[10]
Breast cancer	Fresh frozen, FFPE tissue	Breast cancer subtypes show different histone H3 marks	First protocol to analyze histone PTMs from FFPE tissues	LC–MS, DDA	Super-SILAC	[49]
	Fresh frozen, FFPE tissue	Breast cancer subtypes show different histone H4 marks	Improvement in digestion approaches for the analysis of histone H4 PTMs	LC–MS, DDA	Super-SILAC	[51]
	Fresh frozen, FFPE tissue (LMD)	Breast cancer subtypes show different histone H3 marks	Analysis of histone PTMs from 500,000 laser microdissected cells	LC–MS, DDA	Super-SILAC	[45]
	Fresh frozen, FFPE tissue (LMD)	Differences in histone PTM patters in heterogeneous tumor areas, and in lymphocytes inside/outside the tumor	Analysis of histone PTMs from 1000 laser microdissected cells	LC–MS, DDA	Super-SILAC	[46]
	Fresh frozen, FFPE, OCT (LMD)	Lower levels of histone H1 variants in triple-negative breast cancer samples with worse prognosis	Label-free method to quantitate histone H1 variants from low-abundance clinical samples	LC–MS, DDA	Label-free	[84]
Acute myeloid leukemia (AML)	Frozen primary cells	Differences in H3K9me3 in acute myeloid leukemia patient cells	Targeted analysis of 61 histone PTMs from 1000 primary human cells	LC–MS, targeted	Label-free	[47]
Malignant peripheral nerve sheath tumor	FFPE tissue	Loss of PRC2 results in increased levels of H3K27Ac and H3K36me2 and global loss of H3K27me2/3		LC–MS, DDA/DIA	Label-free	[50]
Cancer	Frozen serum	Detection of histones from circulating nucleosomes by MS	Development of an enrichment method to isolate circulating nucleosomes	LC–MS, DDA	Label-free	[53]
Colorectal cancer	Frozen plasma	Upregulation of several histone PTMs in the plasma of CRC patients	Development of an enrichment method to isolate circulating nucleosomes	LC–MS, DDA	Heavy spike-in peptides	[52]
Hepatocellular carcinoma	Fresh frozen tissue	H4K20me2 and H4K16ac are biomarkers of microvascular invasion in hepatocellular carcinoma		MALDI-IMS	Label-free	[98]
Pediatric brain cancers	FFPE tissue	The missense histone mutation K27M causes changes in H3K36 methylation		LC–MS, DDA	Label-free	[90]
Neurologic dysfunction	Primary lymphoblasts and fibroblasts	Aberrant histone PTM patters in patients with germline mutations in genes encoding for histone H3.3 with progressive neurologic dysfunction and congenital anomalies		LC–MS, DIA	Label-free	[91]
Alzheimer's disease (AD)	Fresh frozen temporal lobe	Increase in H3K27ac and H3K9ac in AD patients	First integrated multi-OMICs approach including MS-based analysis of histone PTMs	LC–MS, DIA	Label-free	[16]
	Fresh frozen temporal lobe	Decrease in histone acetylation in AD patients	SRM method to analyze histone PTMs	LC–MS, DDA, SRM	TMT	[106]
	Fresh frozen frontal cortex	Changes in several histone PTMs in AD patients	MRM method to analyze histone PTMs	LC–MS, DDA, MRM	Label-free	[107]
Septic shock	Frozen plasma	H3 and H2B are elevated in septic shock patient sera		LC–MS, MRM	Heavy spike-in peptides	[55]

^1^FFPE: formalin-fixed paraffin-embedded; OCT: optimal cutting temperature; LMD: laser microdissection. ^2^LC-MS: liquid chromatography–mass spectrometry; DDA: data-dependent acquisition; DIA: data-independent acquisition; MALDI-IMS: matrix-assisted laser desorption/ionization-imaging; SRM: single reaction monitoring; MRM: multiple reaction monitoring. ^3^ Stable isotope labeling by amino acids in cell culture; TMT: tandem mass tag. Ref: reference

## MS-based analysis of histone PTMs and variants in clinical samples

MS is an analytical tool that allows measuring the molecular weight (or, more precisely, the mass/charge ratio, or m/z) of ionized molecules. For peptides and proteins, ionization is achieved mainly by electrospray ionization (ESI) or matrix-assisted laser desorption ionization (MALDI). ESI involves the generation of an electrically charged spray (electrospray) through high voltage [29], while in MALDI ions are generated by laser irradiation of the samples mixed with an energy-absorbing matrix [30]. ESI is usually employed in a liquid chromatography (LC)-MS setup, where the samples are separated by reversed-phase chromatography prior to MS analysis, to reduce their complexity. When applied to histones, MS allows determining the presence of variants differing in only a few amino acids or mutations and can detect the presence of a PTM by measuring a “delta-mass” between the theoretical and experimental m/z of peptides and proteins. This unique capability provides several advantages compared with the antibody-based methods traditionally used to analyze histones in a clinical sample.

First, the identification of a PTM by MS does not require previous knowledge of the type or site of the modification, as antibody-based methods do, and theoretically allows the detection of any PTMs or PTM combination in a single run. In addition, MS approaches allow an accurate quantitation of histone PTMs and variants, which is difficult to achieve using antibodies due to poor signal linearity. Finally, MS overcomes other limitations of antibody-based methods, which include the difficulty in distinguishing closely related sequences (such as those belonging to histone variants), cross-reactivity, and epitope masking, namely the masking of a modification when another is present in a nearby residue. Thanks to these features, MS is currently the method of choice for the analysis of histones, their variants and their PTMs.

**Fig. 2:**
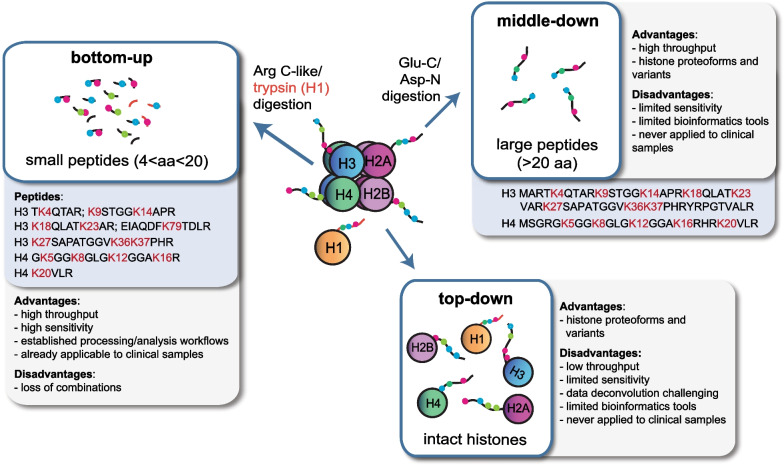
Epi-proteomics approaches for histone analysis. Scheme summarizing the three main MS-based approaches applicable for histone PTM and variant analysis. aa: amino acid

Three main epi-proteomics approaches can be used to investigate histones by MS (Fig. 2). “Top-down” approaches involve the analysis of intact histones, which are usually first chromatographically separated and then MS-analyzed, providing information on the complete panel of histone proteoforms (defined as the different molecular forms in which the protein product of a gene can be found [31]) and variants present in a sample, as well as their stoichiometry. When analyzing whole histones, the number of species with the same molecular weight, but with a different pattern of modifications increases exponentially, thus making their discrimination very challenging and demanding complex custom software which has yet to be developed. In addition, top-down approaches have typically low sensitivity. In “middle-down” approaches, rather long histone peptides (> 5 kDa), usually encompassing most of the N-terminal tails of core histones, are generated through digestion with proteases recognizing residues that occur with low frequency. For example, by cutting at the N-terminus of aspartate, AspN generates the histone H4 1–24 peptide, while GluC cuts at the C-terminus of glutamic acid and can be used to generate histone H3 1–50 peptide. These long peptides contain the entire N-terminal tails of histone H3 and H4, where most of the known and functionally characterized PTMs are localized. Middle-down approaches allow studying combinatorial associations and variants, while at the same time mitigating the issues related to top-down analysis.

The “bottom-up” approach is undoubtedly the most employed for histone analysis. It involves histone digestion into 4–20-amino-acid-long peptides prior to MS analysis. Bottom-up MS can provide information about co-occurring modifications only for nearby residues, thus losing most of the combinatorial information about distant marks. Its strengths, however, lie in its flexibility and the availability of well-established protocols for histone extraction, MS acquisition and analysis, which have been recently applied also to patient-derived samples. On the contrary, the use of top-down and middle-down approaches has been limited to cultured cells and animal tissues so far. By leaving the reader to more technical and general reviews about histone analysis by MS ([32, 33]), in the next sections we will describe the MS-based approaches that have already been applied to clinical samples, highlighting their contribution to our current understanding of cancer epigenetics.

### Histone extraction and enrichment from clinical samples

**Fig. 3:**
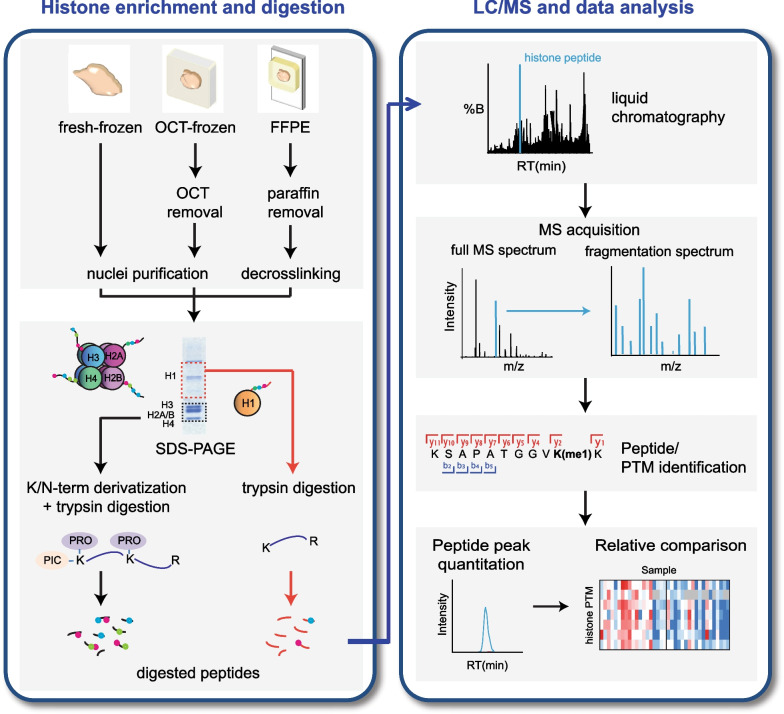
Schematic bottom-up workflow for MS-based analysis of histone PTMs and variants. Histones are enriched through specific protocols from different types of clinical samples, separated by SDS-PAGE and in-gel digested. Digested peptides are separated by liquid chromatography and acquired in the mass spectrometer. MS spectra are then used for peptide identification and quantification, and PTM assignment

Most of the studies investigating histones in disease that have been performed so far employed cell lines, with the aim to identify epigenetic biomarkers [34–36], or to investigate epigenetic mechanisms, especially in connection with the histone-modifying enzyme levels [37–40]. Cell lines are a convenient model system, as they are easy to obtain and to grow, and can be easily manipulated. In addition, they can be obtained in the large amounts needed to generate pure histone preparations, through protocols typically involving nuclei isolation followed by extraction in strong acids (HCl or H_2_SO_4_) [41]. However, despite their advantages, cell lines do not represent ideal models for epigenetic studies, as an extensive and time-dependent epigenetic rewiring, both at the DNA methylation and at histone PTM levels, occurs during the transition from tissue to cell culture [42, 43]. When available, patient-derived samples represent the optimal choice for clinical investigations. Thanks to recent advances in sample preparation, the MS-based analysis of histone proteoforms can be carried out from all the tissue types that can be found in hospital biobanks, namely formalin-fixed paraffin-embedded (FFPE), optimal cutting temperature (OCT)-frozen and fresh frozen tissues (Fig. 3, left panel) [44]. Simplified protocols also exist for low-abundance samples and allow profiling the most common histone PTMs and the somatic histone H1 variants from as low as 1000 cells ([45–47] and paragraph "Low-abundance samples").

Histone extraction protocols from frozen tissues include removal of OCT, when present, followed by nuclei extraction. To minimize sample loss, the acidic extraction steps employed for histone enrichment from cells are usually avoided, or, alternatively, acidic extraction can be performed on whole cell extracts [47]. For FFPE samples, paraffin is removed, and then proteins are extracted and de-cross-linked through incubation at high temperature in the presence of strong detergents [48–50]. Formalin-fixation and paraffin-embedding is the most widely used storage method for clinical specimens, and FFPE tissues represent the most accessible patient tissue for retrospective studies. Although they had been long considered inaccessible for proteomics and PTM studies, due to the extensive protein cross-linking caused by formaldehyde fixation, the analysis of both global proteome and histone PTMs from FFPE tissues is now possible. However, some artefactual modifications persist. For instance, it has been shown that while the levels of most histone PTMs are comparable in FFPE and frozen tissues [49, 51], few methylations and formylations significantly and systematically increase in FFPE tissues. These include an increase in H3K18me1 and H3K79me1/me2 in samples stored for up to 7 years [48], and H3K4me2 in samples stored for 10 years or more, as we recently discovered (unpublished results).

Finally, histone PTMs can be analyzed from circulating nucleosomes present in blood, which represents an attractive source of noninvasive biomarkers. Several ELISA assays have been used to profile specific panels of histone PTMs with potential diagnostic value in colorectal and pancreatic cancers [14, 15]. As an evolution of these ELISA assays, a method based on immunoprecipitation of intact circulating nucleosomes followed by LC/MS analysis has been recently proposed and applied in a proof-of-concept study to compare the histone PTM pattern of healthy subjects and colorectal cancer patients [52]. As previously reported, higher levels of histones were found in the sera of tumor patients, and several histone PTMs, including H3K9 and H3K27 methylation, histone H3 acetylation and histone H2A1R3 citrullination, increased in plasma from cancer patients compared to healthy controls. An alternative method to isolate circulating nucleosomes from serum involves two acid extraction steps [53]. Interestingly, some of the histone PTM changes detected in circulating nucleosomes in tumor compared with healthy subject reflected changes also found in the tissues [52, 53]. These results suggest that cancer patient sera may be a source of epigenetic biomarkers for patient stratification, and indicate that circulating nucleosomes reflect some of the epigenetic features of cancer tissues. However, a systematic investigation of the extent to which circulating nucleosomes are representative of the cancer tissue is still missing. The levels of circulating nucleosomes can also be informative of several pathologies. Elevated levels of circulating histones have been linked with cancer, particularly at advanced stages, but their clinical utility is limited by the lack of specificity of this increase, which can be observed in other diseases [54]. Targeted MS acquisition methods (see paragraph "Low-abundance samples") have been applied to plasma for the quantification of histone H3 and H2B in septic shock patients, which were found elevated compared with control subject and could represent early biomarkers of septic shock [55].

### LC/MS analysis of histone PTMs

Figure 3 schematizes a typical epi-proteomics bottom-up workflow for the analysis of histone PTMs and variants, which can be applied to clinical samples. Once the histones have been purified from the sample of interest, they are digested into peptides prior to MS analysis. When dealing with clinical samples, histone digestion is usually performed in-gel, which serves two purposes: (1) removing the MS contaminants (e.g., OCT, detergents) present in the histone preparations; and (2) enriching histones from often crude extracts, by allowing the excision of a single gel band. The only protease that works efficiently in-gel is trypsin, which is appropriate for histone H1 analyses, but not for core histones. Indeed, trypsin generates core histone peptides that are too short for MS analysis, due to the high number of lysines and arginines present in their sequences. In addition, because of poor cutting efficiency next to modified residues, trypsin generates peptides of variable length, whose quantification is difficult. These issues can be overcome by using trypsin in combination with the chemical derivatization at lysine residues with acylating agents, thus blocking trypsin digestion at lysines and producing peptides of appropriate length for MS analysis. The most common acylating agent is propionic anhydride (PRO) [56], but others have been employed and can be advantageous in specific contexts (e.g., to study physiological propionylation) [57, 58]. A second round of derivatization of the digested peptides, usually with propionic anhydride or phenyl isocyanate (PIC), further increases peptide hydrophobicity and detectability, particularly for short and hydrophilic peptides ([59]). Recently, the PRO-PIC approach (lysine propionylation combined with N-terminal derivatization with PIC) has been shown to be the most successful strategy in terms of number of modified peptides quantifiable and starting amount needed [46]. As an added advantage, derivatization approaches also improve the discrimination of isobaric peptides, by causing chromatographic retention time shifts that can help their quantification [58].

Following digestion, histone peptides are separated by reversed-phase high-performance liquid chromatography (HPLC). Besides reducing the complexity of the sample prior to MS analysis, an efficient chromatographic step is instrumental for the separation of isobaric species, namely peptides with the same mass, but with different sequences. For instance, peptides containing one methylation on either H3K27 or H3K36, which fall within the same proteolytic peptide, can be distinguished because they elute from the chromatographic column with slightly different retention times. The chromatographic performance and reproducibility are therefore crucial during histone PTM MS analysis. At the tip of the chromatographic column, peptides are ionized by ESI, and the ions are injected into a high-resolution mass spectrometer, where their m/z are measured.

In “data-dependent acquisition” (DDA) routines, the most abundant ions are then broken into smaller fragments, whose m/z is also acquired. The output of this analysis is represented by “full MS spectra” (or MS1 spectra), showing the m/z of the intact peptides, and “fragmentation spectra” (or MS2 spectra), where the m/z of the fragments are displayed (Fig. 3). Such experimentally determined spectra are then matched to the theoretical spectra present in a database, to obtain the mass and the sequence of the peptides, as well as the presence and location of modifications (Fig. 3). While DDA routines are widely used and represent a well-established strategy to analyze abundant modifications, they suffer from an intensity bias that can limit the detection of less abundant PTMs. Data-independent acquisition (DIA) approaches (reviewed in [60]) involve the fragmentation of all the ions within a given m/z window, overcoming intensity biases. Another advantage of DIA is the possibility to use both MS1 and MS2 spectra for quantitation, helping the discrimination of isobaric and co-eluting peptides [61, 62]. In the context of clinical samples, DIA was used to investigate histone PTM changes due to loss-of-function alterations in polycomb-repressive complex 2 (PRC2), which contains the H3K27me3-specific methyltransferase EZH2, in malignant peripheral nerve sheath cancer FFPE samples. This analysis showed that loss of PRC2 causes a decrease in H3K27me3 and an increase in H3K36me2 and H3K27ac [50]. It was also used in a multi-OMICs analysis of epigenetic alterations associated with AD [16].

MS-based quantitation strategies (reviewed in [32]) typically involve the extraction of the peaks matching the m/z value and chromatography retention time of the histone peptides from the chromatographic profile. Such peaks are known as eXtracted Ion Chromatograms (XICs) (Fig. 3), and can be obtained manually or through dedicated software, EpiProfile [63, 64]. XICs can be directly compared across samples that were separately acquired in label-free analyses, a strategy that was employed to profile histone PTMs in Posterior fossa A ependymomas [65]. Alternatively, samples can be mixed with an internal standard to which the XICs are compared, to improve the quantification accuracy. Labeled histones to be used as internal standard can be generated by growing one or more cell lines in media containing isotope-encoded amino acids, using the Stable Isotope Labelling by Amino acids in Cell culture (SILAC) strategy [66–68]. A SILAC-based spike-in approach has been applied to clinical samples to profile histone PTM patterns in normal and tumor tissues [10], revealing tumor-specific and subtype-specific changes, as well as a generalized decrease in H3K14ac [10, 51]. A comparison of breast cancer molecular subtypes using the same approach also showed different epigenetic patterns, which included a decrease in H3K27me3, and an increase in H3K9me and H3K36me1/me2 in the aggressive triple-negative subtype [45, 49]. As an alternative to SILAC-labeled histones, synthetic isotope labeled peptides could be used, with the advantage of allowing an absolute quantitation [69]. In addition, synthetic peptides do not have trace amounts of unlabeled peptides as found in SILAC extracts (unpublished results), which can interfere with the analysis of extremely low-abundance samples.

### LC/MS analysis of histone variants and oncohistones

Histones are highly conserved, and each histone class is encoded by a cluster of separate genes known as variants. Histone H3 has 5 variants, histones H2A and H2B have 20 and 17 variants, respectively, and H4 has one [70]. While “canonical” core histones are deposited on chromatin in a replication-dependent manner, histone variants, which are encoded by different genes, are expressed throughout the cell cycle [2, 71]. Core histone variants display different degrees of similarity compared with their canonical counterparts and can show different expression patterns and PTMs and be enriched at specific genomic regions. Linker histone H1 also exists in multiple variants (11 in human and mouse), which can bind differently to the nucleosome and contribute to generating different higher-order chromatin structures that, in turn, affect nuclear functions (reviewed in [72]). Aberrations in the deposition of core and linker histone variants have been extensively described in tumors (reviewed in [23] and [73]). In addition, recurrent histone mutations have been identified in different types of cancer (reviewed in [73, 74]). The most famous example is the H3.1/H3.3K27M missense mutation, which was originally discovered in pediatric high-grade gliomas [75–79] and was later identified in other pediatric tumors [80–82]. These mutant histones, usually referred to as “oncohistones,” impair the binding of histone methyltransferases and display a dominant-negative effect, resulting in a decrease in methylation levels, despite the fact that they usually represent a minor portion of the histone pool [74, 83].

MS-based strategies offer a particularly useful tool for the analysis of histone variants and mutations, as their frequently limited sequence differences make challenging their detection through antibodies. The ability to quantify individual histone variants depends on the existence of proteolytic peptides that can be analyzed by MS. Because their sequences are more divergent, histone H1 variants can be reliably quantified using standard bottom-up approaches based on trypsin digestion (Fig. 3), as well as top- and middle-down methods. A bottom-up label-free workflow specific for the analysis of somatic histone H1 variants was recently implemented and applied to triple-negative breast cancer patient samples, showing a general decrease in histone H1 in tumors from patients who relapsed after chemotherapy, compared to those with better outcomes [84]. MS approaches can also provide information on histone H1 PTMs [85–87].

Core histone variants can also be investigated by MS (reviewed in [88]). Bottom-up methods allow distinguishing histone H3.3 from H3.1 thanks to an amino acid difference (amino acid 31) falling in a peptide detectable by bottom-up MS (peptide 27–40) [89]. The H3K27M mutation also falls in the same tryptic peptide and has been investigated by bottom-up MS in pediatric glioblastoma [90]. Germline mutations in genes encoding for histone H3.3 have also been associated with a phenotype of neurodegenerative disorder characterized by developmental delay, where the levels and histone PTM profile of H3.3 were investigated by MS [91]. However, because of the small size of bottom-up peptides, many variants—for instance histone H2B variants—cannot be distinguished. To solve this problem, histones can be chromatographically separated prior to MS acquisition [88], or, alternatively, top- and middle-down MS can be used [92–95]. These approaches, however, have only been performed so far in cell lines or mouse tissue [95, 96].

### MALDI-MS imaging

**Fig. 4:**
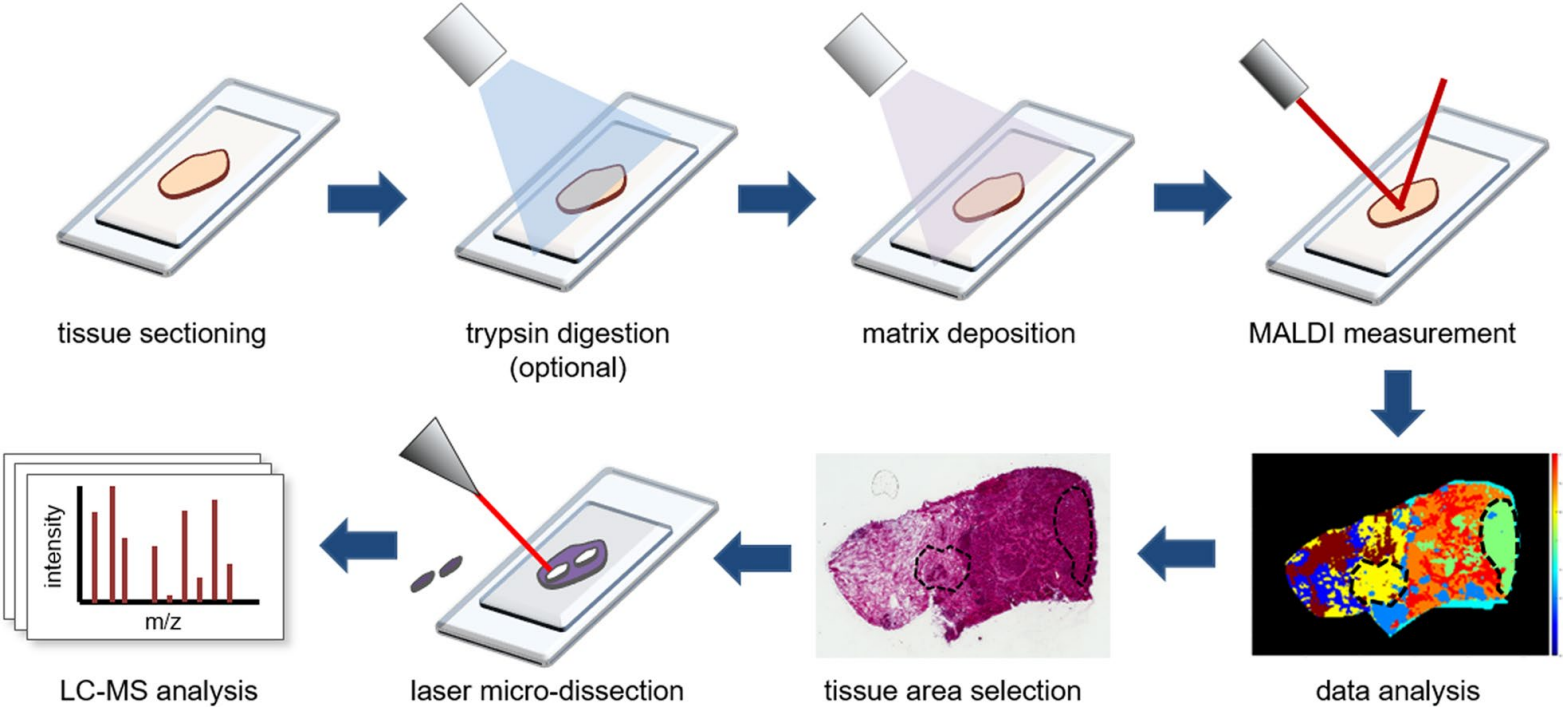
MALDI imaging workflow. Tissue sections are mounted on slides appropriate for MALDI imaging, are digested with trypsin (as an optional step) and covered with a MALDI matrix. The tissue slides are scanned by a laser beam, which generates MS spectra for each xy coordinate. MALDI imaging profiles can guide the selection of area of interest to be laser microdissected and analyzed by LC/MS

Although LC/MS methods are by far the most widely used for the MS-based analysis of histones, their main disadvantage consists in the loss of spatial information. This issue can be partially overcome by selecting specific tissue areas by laser microdissection, but a much more detailed and comprehensive spatial view can be obtained by MALDI imaging [97]. As the name suggests, this approach involves the use of a MALDI source. The MALDI matrix is directly sprayed on the tissue, which is then scanned by a laser beam, generating MS spectra for each measured tissue spot (Fig. 4). By plotting ion intensities as a function of their x and y coordinates within the tissue sections, spatial expression maps are generated for every ion. Thus, MALDI imaging allows obtaining spatial information similarly to immunohistochemistry, with the advantage of being applicable simultaneously to hundreds of peptides. Proteins can be analyzed in a top-down manner, or after *in situ* proteolytic digestion. When dealing with FFPE tissues, proteins must be digested into peptides, and deparaffinization/de-cross-linking steps are required. OCT-frozen tissues, on the other hand, are not compatible with the MALDI imaging approach, as OCT is a strong MS contaminant.

Histone PTMs/variants were identified as differentially regulated in several MALDI imaging global proteomics profiling studies. For instance, MALDI imaging analysis of intact proteins in hepatocellular carcinoma tumors revealed an association between microvascular invasion—which is associated with tumor recurrence and postoperative mortality—and an increase in histone H4K16ac and K20me2 [98]. A similar workflow was also used to visualize changes in histone acetylation levels during treatment with an HDAC inhibitor in a mouse model of gastrointestinal cancer [99]. Ultra-high mass resolution MALDI imaging was employed to spatially profile intact proteins in a mouse model of glioblastoma and resolved many core histone PTMs and variants [100]. Thanks to the improved resolution of this approach, for the first time differentially methylated histone proteoforms could be distinguished, in addition to differentially acetylated forms. A top-down workflow optimized specifically for the detection of histones was also developed and applied to analyze the distribution of histone H1 variants in mouse brain [101].

Despite many advantages, MALDI imaging has several limitations. The first is the difficulty in assigning an identification to the m/z values that have been measured, due to the poor fragmentation capability of this technique. Another challenge is represented by protein quantification. To overcome these issues, MALDI imaging is usually associated with a parallel LC/MS run. One of the most interesting applications of MALDI imaging is its combination with laser microdissection (Fig. 4). The spatial molecular profiles generated by MALDI imaging can be used to guide the selection of areas of interest by LMD, representing a molecular-driven alternative to the morphological evaluation carried out by a pathologist. Such an approach was recently employed to profile histone PTMs in heterogeneous breast cancer regions. MALDI imaging profiling of lipids was used to define tumor regions characterized by different molecular features, which were then isolated by laser microdissection and subjected to LC/MS analysis to obtain quantitative profiles of histone PTMs, revealing differences in adjacent tumor areas [46]. Although in this case lipidomic profiling was used to define tissue molecular features, ideally, MALDI imaging of histone proteoforms could be performed upstream of laser microdissection to define regions characterized by distinct epigenetic profiles.

## Challenges and perspectives

### Middle- and top-down approaches

While bottom-up MS approaches offer efficient sequencing and are sufficiently high throughput for complex samples, which allowed the development of protocols applicable to patient-derived tissues, they are associated with a loss of information about combinatorial PTM patterns and variant differences. Indeed, bottom-up methods only allow quantifying the frequency of coexistence of very close residues, such as H3K9-K14, H3K18-K23, H3K27-K36 and H4K5-K8-K12-K16, missing long-range interactions. This type of information can be obtained by top-down and middle-down workflows, whose application to clinical samples remains, however, very challenging. As a compromise between top-down and bottom-up approaches, middle-down has become increasingly more popular during recent years and has witnessed encouraging progress, from both the experimental and data analysis points of view (reviewed in [102]). Nevertheless, the reproducibility and robustness of middle-down workflows are still limited compared to bottom-up [102]. Middle-down experiments require dedicated instrument setups, where histone tails are separated through weak cation exchange columns, and specific fragmentation methods optimized for highly charged peptides must be used. The biggest challenge of middle-down workflows is, however, data analysis and interpretation. The layers of information generated by middle-down, which include coexistence frequency of combinatorial PTMs, on the one hand represent precious information, but on the other demand complex deconvolution methods, which only a few laboratories worldwide are expert in.

Another issue of middle-down approaches is related to sample preparation from clinical specimens. Most of the histone preparation protocols used so far for the analysis of histones involve SDS-PAGE separation, which enriches histone from crude total protein or nuclear extracts prior to digestion, and eliminate MS contaminants. However, only the trypsin protease functions efficiently in-gel. As an alternative to SDS-PAGE separation, MS contaminants can be removed by acetone precipitation, and histones can be enriched through a C18 micro-column [51]. This approach would be compatible with in-solution digestion with the proteases used in middle-down approaches, but requires higher starting amounts of material, which may be difficult to obtain in the case of clinical samples. These challenges will have to be solved in order to apply middle-down approaches to patient-derived samples.

### Low-abundance samples

An important improvement achieved during the last years is the adaptation of the protocols developed for histone extraction from FFPE, fresh frozen and OCT-frozen samples to manually macrodissected [45] and laser microdissected tissues [45, 46]. These approaches enabled significantly scaling down the starting amounts needed for histone PTM and variant analysis. Using a classical DDA approach combined with a spike-in standard, all the most common methylation and acetylations (including the differentially modified forms of histone H3 peptide 27–40) can be comprehensively profiled from 100,000 cells, while 35 and 40 differentially modified peptides can be quantitated from tissue areas dissected from FFPE and OCT-frozen sections, respectively, containing as low as 1000 cells [46]. From the same amounts of cells, all the somatic histone variants can also be analyzed using a label-free approach [84].

As an alternative to label-free and spike-in methods, chemical labeling quantitation approaches can be employed. Two options that have gained popularity in the last years for global proteomics studies, and that could be particularly useful for the analysis of histones in clinical samples, involve the chemical modification of peptides following digestion, using isobaric tags for relative and absolute quantification (iTRAQ) [103] or tandem mass tags (TMT) [104]. These strategies employ tags that share the same chemical structures and mass, but can be distinguished at the MS2 level thanks to the presence of isotopes substituted in different regions of the tag. Up to 16 samples can be differentially labeled and combined for MS analysis, thus increasing sample throughput and multiplex capabilities, and decreasing acquisition time. TMT labeling was used to profile histone acetylations in the temporal lobe of control human subjects and patients affected by Alzheimer's disease [105, 106]. Because TMT/iTRAQ labels are isobaric, the differentially labeled peptides from the different conditions elute together and are analyzed simultaneously in the mass spectrometer as one ion peak during MS1, improving the analysis of low-abundance samples.

Furthermore, as an alternative to DDA (and DIA) acquisition, targeted MS methods, which include single ion monitoring (SIM) and single-, multiple- and parallel-reaction monitoring (SRM, MRM and PRM, respectively), can be used to analyze with higher sensitivity and throughput previously defined peptides of interest. In addition, targeted workflows are more readily applied in a clinical setting. Indeed, while DDA/DIA acquisition routines are useful tools to investigate histone PTM/variant levels in clinical samples during a discovery phase, they require specific equipment, as well as complex processing and analysis workflows that are unlikely to be translatable to the clinical routine in the near future. SRM and MRM, instead, require MS instrumentation often already used in hospitals for other applications. SRM has been applied to study histone acetylations in the brain of AD patients, revealing a significant decrease in acetylation in the temporal lobe of Alzheimer’s patients compared with aged controls [106]. PRM was also used to investigate histone PTMs in AD, identifying a decrease in H2B K108me1 and H4R55me1, and an increase in H2B K120ub in diseased patients [107]. MRM workflows have been developed and applied to the quantification of 42 differentially modified histone peptides [108], or specifically to H3K56ac [109]. The increased sensitivity provided by MRM approaches can be particularly advantageous for low-abundance samples. A recent targeted assay allowed the detection and quantitation of 75 histone peptides from 10,000 cells, 61 from 1000 primary human stem cells, and 37 from 1000 AML patient cells [47]. The profiling of AML patient specimens revealed a change in H3K9me3 levels between two subsets of AML samples, which was paralleled by changes in the gene expression levels of SETDB1, one of the methyltransferases responsible for the trimethylation of H3K9 [47]. This last study sets an important milestone for the applicability of MRM approaches to histone PTM analysis in clinical samples, by demonstrating the possibility to apply it to patient-derived samples available in very limited amounts. Issues could potentially arise from the analysis of tissue samples (particularly FFPE), which require more processing steps and produce more crude extracts.

Stable isotopic labeled peptides are often used as internal standards in MS-targeted approaches to measure the absolute peptide concentrations, an aspect that would be extremely useful for the quantitation of biomarkers in a clinical setting. Another recent technological implementation concerns the use of direct injection MS, as an alternative to HPLC separation, to analyze 200 histone PTMs with a 1-min acquisition time [61]. This workflow would solve reproducibility issues often linked with nano-HPLC separation and provide a throughput potentially allowing the analysis of 1000 samples per day. Although the applicability of this workflow to patient-derived clinical samples has still to be verified, its combination with targeted MRM acquisitions would represent an ideal workflow to routinely process samples in the clinic. Altogether, the methodological advances in the MS-based analysis of histones samples represent an important step toward the investigation of histone proteoforms in low-abundance samples, which include early cancer lesions, micrometastases, specific tumor areas and nucleosomes isolated from blood. They also open the way for the investigation of histones in the context of tissue heterogeneity, enabling the analysis of specific morphological structures, cell types or tumor areas within the same tissue section. Nevertheless, the current achievements are far from the single-cell resolution reached by other -omics technologies. Such resolution, which is becoming within reach for whole proteomics studies [110], appears still extremely challenging to obtain and far in the future for PTMs, given their low stoichiometry and the complexity associated with their analysis.

### Uncommon modifications

As of 2015, more than 500 histone PTMs were cataloged [111]. The library of histone modifications is constantly expanding, with the discovery of new chemical groups able to bind to various amino acid residues of histones. Historically, researchers mostly focused on the most abundant histone marks (mainly lysine methylation and acetylation), although a large number of diverse PTMs have been reported to occur on histones, and could be potentially interesting from the clinical point of view. For instance, malonylation was found to be increased in type 2 diabetes mouse models [112] and in human embryonic brains with diabetes‐induced neural tube defects [113]. In the last three years, five novel histone PTMs were characterized: histone glycation [114], benzoylation [115], serotonylation [116], lactylation [117] and dopaminylation [118]. Of these, lactylation and glycation may be particularly relevant in the context of disease. Lactate and other glycolytic by-products as glyoxal and methylglyoxal accumulate in tumor cells as a consequence of the Warburg effect and are able to react with histones. Lactylation is catalyzed by P300 and directly stimulates gene transcription. Histone glycations represent a non-enzymatic reaction between the amino group of lysine or arginine and a carbonyl of a reducing sugar that rearrange to form advanced glycated end products (AGEs), that have been involved in cancer and diabetes [119]. It has been shown that in vitro AGEs drive DNA–histone and histone–histone cross-linking that can disrupt both nucleosome assembly and chromatin accessibility [120]. In addition, glycation induces histones code deconstruction, triggering senescence [121].

The analysis of all the PTMs potentially occurring on histones is challenging for several reasons [122]: (1) the large diversity of PTMs that can occur on histones; (2) the high number of amino acid residues on which they can occur; (3) the difficulty in distinguishing isobaric PTM combinations (e.g., the mass of the acetyl group (42.0106 Da) is equal to the sum of the masses of a methyl (14.0157 Da) and a formyl groups (27.9949 Da); (4) potential masking by isobaric histone amino acid variations (e.g., the serine to threonine substitution has the same delta mass of a methyl group). Thus, it is necessary to profile several histone PTMs at once. Standard database search strategy implemented in popular tools such as Mascot [123], SEQUEST [124], and Andromeda [125] can handle only a few variable modifications. This is because allowing for multiple possible modifications leads to a combinatorial expansion that dramatically increases the search space, estimated to be in the order of millions. For example, the currently known modifications on histone H4 peptide 4–17 (six occurring on K5, six on K8, eight on K12 and six on K16 [126]) yield 3087 possible combinations for only one peptide. This search space explosion causes an exponential increase in the search time, since all possible modified forms of each peptide must be considered by the software, and increases the probability of incorrectly assigning a PTM, leading to a higher prevalence of false identifications [127]. The presence of false-positive identification compromises the capability of search engines to distinguish true from false-positive matches, leading to a significant reduction in the number of identified spectra at a given false discovery rate (FDR) [127]. Within this context, several bioinformatics tools known as “blind” or “open” search have been developed, which allow searching for all known and possibly even unknown PTMs at once. Recent examples are MSfragger [128] and Open-pFind [129]. These approaches allow wide precursor mass error tolerances of hundreds of Daltons and use strategies such as fragment ion indexing and sequence tags [130] to deal with the search space explosion; allowing including any PTM in the fragment spectrum evaluation. Open search algorithms have been employed to identify previously unknown PTMs in diseases [131, 132]. However, one of the weaknesses of such algorithms is modification localization [133]; as a consequence, they are not generally tailored to be applied to hypermodified proteins such as histones. Nevertheless, one successful application of an unrestrictive search algorithm in the context of histones was reported [134], which allowed the identification of two novel histone marks, tyrosine hydroxylation and lysine crotonylation.

Another open issue is represented by missing values. The above-described uncommon modifications are often low-abundance and difficult to be reliably quantitated in multiple samples. This issue also applies to common modifications in low-abundance samples. While common histone PTMs are usually present in all sample types, they may fall under the limit of detection when dealing with limited sample amounts (for instance, this is the case of PTMs on H3K27 and H3K36, as described in [46]). For analyses that cannot deal with missing values, computational imputation methods can be used. Alternatively, the problem of missing values may be overcome at the experimental level by using either targeted or DIA acquisition methods, which are not affected by the intensity bias of DDA. Because in DIA acquisition all the ions are fragmented regardless of their intensity, DIA is much less dependent on both the amount of starting material and PTM abundance.

### Investigating epigenetic mechanisms linked with histone aberrations

Given the complexity of the mechanisms involved in epigenetic regulation (Fig. 1), investigating the causes of the histone aberrations identified by MS and their downstream effects is not an easy task. In the simplest scenario, histone PTM changes are determined by aberrations in the levels of the corresponding HME. For instance, a correlation exists between the increase in H3K9me3 and the upregulation of several methyltransferases acting on the H3K9 observed in tumors compared with normal tissues [10]. However, a correlation between histone PTM/HME levels is not always observable. This is for instance the case of the hallmark reduction in histone H4K16ac and H4K20me3 in tumors [9, 135]. Loss of H4K16ac was associated with diminished recruitment of the acetyltransferases MOZ, MOF and MORF at repeated sequences [9, 136], while a correlation between H4K20me3 and its HMEs has not been clearly demonstrated [137]. As another example, in most cases H3K27 methylation does not correlate with EZH2 levels [138]. One exception is melanoma, where levels of both H3K27me3 and EZH2 were found increased, and silenced transcription of the tumor suppressor genes E-cadherin and RUNX3 [139]. Additional factors relating to the level of histone PTMs include an altered function of HMEs, or of multi-subunit complexes to which they belong, differences in proliferation rates [10], and potential inter-dependence of histone PTM and DNA methylation levels [140–142]. Changes in expression levels and/or mutations of histone chaperones can also influence histone PTMs and variants [7]. Additionally, spontaneous non-enzymatic reactions mediated by chemically reactive metabolites can modify histones, and the metabolic condition of the tumor cells can influence histone PTM levels (reviewed in [143]). For instance, the hypoxic metabolism of posterior fossa A ependymoma generates intermediary products that favor higher levels of H3K27ac (acetyl-CoA) and lower levels of H3K27me3 (*α*-ketoglutarate, which stimulates the activity of the H3K27 demethylases KDM6A and KDM6B)[65]. In the same tumor type, a protein containing a peptide that mimics the oncohistone mutation K27M (EZHIP), inhibits the H3K27 methyltransferase activity of PRC2, providing another mechanism explaining H3K27me3 low levels in PFA ependymoma [144].

The downstream consequences of histone aberrations can also be studied, which requires the integration of MS data with other -OMICs technologies. First, ChIP-seq experiments are necessary to relate bulk quantitative MS information with the genomic distribution. The availability of specific and reliable antibodies may represent a particularly relevant problem for less characterized and novel modifications, for which reagents are not available. Further integrating ChIP-seq information regarding changes/aberrations in the genomic distribution of histone PTMs and variants with transcriptomic, proteomics and metabolomics profiling can provide a global vision on the phenotypic consequences of epigenetic alterations. A remarkable example of a multi-OMICs approach applied to the investigation of epigenetic features was reported in the context of AD [16]. Based on RNA-seq evidence that the CREBBP and EP300 histone acetyltransferases were upregulated in postmortem AD brains, the authors set to profile by MS histone PTMs, finding an increase in several histone acetylations, including H3K27ac and H3K9ac. ChIP-seq analyses of these marks revealed changes linked to disease pathways in AD. These findings provided evidence of a reconfiguration of the epigenome in AD.

Because epigenetic changes, unlike genetic alterations, can be reverted, these investigations can uncover potential points of therapeutic intervention at different levels. For instance, modulators of HME activity, many of which have been already developed [145], can be used to restore histone PTM levels, while molecules acting on histone readers (e.g., bromodomain inhibitors [146]) can revert the downstream effects of histone aberrations. Investigating the downstream effects of histone alterations through integration with other -OMICs approaches can also highlight aberrant protein activities or pathways, which could represent additional targets for therapeutic intervention.

## Conclusions

The last 10 years have witnessed important advances in the application of MS-based approaches for the analysis of histones, their PTMs and their variants in clinical samples. The main achievements include the development of protocols for the extraction and enrichment of histones from all the major sources of clinical samples, the scaling down of the starting material required, and the implementation of robust bottom-up workflows for the quantitative profiling of both histone PTMs and histone variants. However, a significant gap still exists between the technological improvements in MS-based technologies for histone analysis and their application to clinical samples. Besides overcoming the technical challenges described in the previous sections, key to the success of this type of experiments will be the integration of MS-based analysis of histones with complementary approaches and different expertise. When aiming at the discovery of epigenetic biomarkers for patient stratification, MS-based technologies used in the discovery phase will have to be translated into clinically applicable assays that can be routinely performed in a high throughput and automatized fashion. As mentioned previously, targeted MRM experiments may serve this purpose, but ideally simple ELISA assays would be more readily usable in a clinical setting. One of the most important advances that we envision for the near future for epigenetic biomarker discovery is the implementation of robust and reproducible workflows for the quantitation of histone PTMs, and possibly variants, from sera. The ability to profile histones in a noninvasive manner would allow the analysis of tumors that are early-stage or difficult to reach (e.g., brain), and open the way for longitudinal analyses. When investigating epigenetic mechanisms linked with histone aberrations, integration with genomics, transcriptomics, proteomics and metabolomics data will be fundamental to dissect the complex epigenetic regulatory mechanisms and to identify novel therapeutic targets to restore/counterbalance epigenetic abnormalities.

Last but not least, the application of MS technologies to clinical samples requires the availability of well-annotated patient-derived samples, whose acquirement often represents a bottleneck of research projects involving human tissues. A close collaboration between researchers, clinicians and pathologists is of utmost importance, both to make precious patient samples available for research purposes, and to ensure that the MS-based technologies serve truly relevant clinical questions.

## Data Availability

Not applicable.

## Abbreviations

AD: Alzheimer disease
DDA: Data-dependent acquisition
DIA: Data-independent acquisition
ESI: Electrospray ionization
FFPE: Formalin-fixed paraffin-embedded
HDAC: Histone deacetylase
HME: Histone-modifying enzyme
HPLC: High-performance liquid chromatography
LC: Liquid chromatography
LMD: Laser microdissection
MALDI: Matrix-assisted laser desorption ionization
MRM: Multiple reaction monitoring
MS: Mass spectrometry
OCT: Optimal cutting temperature
PIC: Phenyl isocyanate
PRM: Parallel reaction monitoring
PTMs: Posttranslational modifications
SILAC: Stable isotope labeling by amino acids in cell culture
SRM: Single reaction monitoring
TMT: Tandem mass tag
XICs: EXtracted ion chromatograms
